# Administration of GDF3 Into Septic Mice Improves Survival *via* Enhancing LXRα-Mediated Macrophage Phagocytosis

**DOI:** 10.3389/fimmu.2021.647070

**Published:** 2021-02-17

**Authors:** Peng Wang, Xingjiang Mu, Hongyan Zhao, Yutian Li, Lu Wang, Vivian Wolfe, Shu-Nan Cui, Xiaohong Wang, Tianqing Peng, Basilia Zingarelli, Chunting Wang, Guo-Chang Fan

**Affiliations:** ^1^Department of Critical Care Medicine, Shandong Provincial Hospital Affiliated to Shandong First Medical University, Jinan, China; ^2^Department of Pharmacology and Systems Physiology, University of Cincinnati College of Medicine, Cincinnati, OH, United States; ^3^Department of Critical Care Medicine, The Second Hospital, Cheeloo College of Medicine, Shandong University, Jinan, China; ^4^Department of Critical Care Medicine, Renmin Hospital of Wuhan University, Wuhan, China; ^5^Division of Critical Care Medicine, Cincinnati Children's Hospital Medical Center, Cincinnati, OH, United States; ^6^Department of Pediatrics, University of Cincinnati College of Medicine, Cincinnati, OH, United States; ^7^Department of Anesthesiology, Beijing Cancer Hospital, Peking University School of Oncology, Beijing, China; ^8^The Centre for Critical Illness Research, Lawson Health Research Institute, London, ON, Canada

**Keywords:** growth differentiation factor 3, macrophage, phagocytosis, sepsis, LXRα, CD5L

## Abstract

The defective eradication of invading pathogens is a major cause of death in sepsis. As professional phagocytic cells, macrophages actively engulf/kill microorganisms and play essential roles in innate immune response against pathogens. Growth differentiation factor 3 (GDF3) was previously implicated as an important modulator of inflammatory response upon acute sterile injury. In this study, administration of recombinant GDF3 protein (rGDF3) either before or after CLP surgery remarkably improved mouse survival, along with significant reductions in bacterial load, plasma pro-inflammatory cytokine levels, and organ damage. Notably, our *in vitro* experiments revealed that rGDF3 treatment substantially promoted macrophage phagocytosis and intracellular killing of bacteria in a dose-dependent manner. Mechanistically, RNA-seq analysis results showed that CD5L, known to be regulated by liver X receptor α (LXRα), was the most significantly upregulated gene in rGDF3-treated macrophages. Furthermore, we observed that rGDF3 could promote LXRα nuclear translocation and thereby, augmented phagocytosis activity in macrophages, which was similar as LXRα agonist GW3965 did. By contrast, pre-treating macrophages with LXRα antagonist GSK2033 abolished beneficial effects of rGDF3 in macrophages. In addition, rGDF3 treatment failed to enhance bacteria uptake and killing in LXRα-knockout (KO) macrophages. Taken together, these results uncover that GDF3 may represent a novel mediator for controlling bacterial infection.

## Introduction

Sepsis is characterized as life-threatening multi-organ dysfunction caused by a dysregulated host response to infection (1, 2). Although aggressive antibiotic treatments are applied to control bacterial infection at the early stage, sepsis remains a leading cause of death in intensive care units (3, 4). Recently, in a post-mortem study of 235 sepsis patients, about 80% of patients had unresolved septic foci at death (5), indicating that sepsis-induced death is closely associated with the failure of the host's immunity to eradicate invading pathogens (6, 7). Hence, it is becoming clear that sepsis is not only the result of excessive inflammation but also is coupled with defective immune system with impaired pathogen clearance function (1). However, there is currently lack of specific pharmacologic therapy that can target the host immune response to eradicate invading pathogens (8, 9), and drug-resistance bacteria are also emerging (10). Thus, it is urgently needed to develop alternative treatment strategies to enhance host defenses for timely clearance of bacteria (11).

Macrophages are the key components of the immune system, forming a bridge between innate and adaptive immunity by producing a myriad of cytokines, phagocytosing invading pathogens, and presenting antigens to other immune cells (12). As professional phagocytic cells, macrophages are vital to the resolution of inflammation through high endocytic clearance capacities and trophic factor synthesis, accompanied by reduced pro-inflammatory cytokine secretion (13). Patients with defects in the phagocytic function of macrophages are predisposed to intracellular microorganisms and typically manifest early dissemination of infection (14, 15). Therefore, to provide more efficacious therapies for sepsis, it would be very significant to better understand macrophage function and the underlying mechanisms that govern phagocytosis.

As a member of TGF-β superfamily, GDF3 was initially identified in stem cells during early embryonic development (16–18). Later work defined it as an adipogenic cytokine regulating adipose tissue homeostasis and energy balance (19–21), and a marker of stem cells (22, 23). Recently, several studies suggest that GDF3 might play a vital role in modulating inflammatory response upon acute sterile injury (24, 25). For example, Varga et al. reported that GDF3 could serve as a macrophage-derived factor in tissue repair during skeletal muscle regeneration (24). Patsalos et al. further demonstrated that *in vivo* administration of GDF3 could compensate for the age-related loss of reparative macrophages, which improved the kinetics of muscle repair following acute sterile injury (25). Furthermore, our recent studies revealed that serum GDF3 levels in septic patients are elevated and strongly correlate with severity of sepsis and 28-day mortality (26). However, macrophages treated with recombinant GDF3 protein (rGDF3) exhibit reduced production of pro-inflammatory cytokines, compared to controls upon endotoxin challenge (26). Therefore, these prior findings promoted us to clarify whether GDF3 could regulate macrophage phagocytic function in a clinically relevant sepsis model.

In this study, we observed that the administration of rGDF3 either before or after cecal ligation and puncture (CLP) surgery significantly decreased bacterial burden and systemic/local inflammation, alleviated multiple organ injury and animal mortality, compared to control mice. Mechanistically, we identified that rGDF3 treatment promoted phagocytosis and bacteria killing activity of macrophages through activation of LXRα pathway. Accordingly, rGDF3-mediated phagocytosis and bacterial killing were abolished in LXRα-deficient macrophages or by pharmacological inhibition of the LXRα pathway using GSK2033 in macrophages. Therefore, our study indicates that GDF3 may play a previously unrecognized role in controlling bacterial dissemination during sepsis.

## Materials and Methods

### Mice

C57BL/6J mice were purchased from Jackson Laboratories (Bar Harbor, ME, USA). The global LXRα-KO mice were a gift from Dr. Basilia Zingarelli (Cincinnati Children's Hospital Medical Center, Cincinnati, OH). All mice were maintained and bred under specific pathogen-free conditions in the Division of Laboratory Animal Resources at the University of Cincinnati Medical Center. All animal experiments conformed to the Guidelines for the Care and Use of Laboratory Animals prepared by the National Academy of Sciences, published by the National Institutes of Health, and approved by the University of Cincinnati Animal Care and Use Committee.

### Polymicrobial Sepsis Model, Survival, and Bacterial Burden Assay

Mouse polymicrobial sepsis was surgically induced by CLP as described previously (27). In brief, to determine whether rGDF3 had any preventive effects *in vivo* against polymicrobial sepsis, recombinant mouse GDF3 Protein (20 μg/kg body weight) (R&D Systems, Cat. # 9009-GD-010) or BSA vesicle was injected into the tail vein of wild-type (WT) mice (8-week old, male) 3 h before CLP. Considering the cyclic hormonal changes during the ovulatory cycle in female mice might cause excess variability, male WT mice were used in the CLP model. Then, mice were anesthetized by intraperitoneal injection of a mixture of ketamine (100 mg/kg) and xylazine (5 mg/kg). A midline laparotomy was then performed. The cecum was exposed and ligated at 1.5 cm from the distal end with a 5–0 sterile silk suture. A single through and through puncture was made at the middle between the ligation and distal end of the cecum with a 21-gauge needle. After puncturing, the cecum was gently compressed to extrude a small amount of cecal content and returned to the abdominal cavity. The abdominal wall incision was closed in layers. After surgery, pre-warmed phosphate buffered saline (0.05 mL/g body weight) was administered subcutaneously. Postoperative pain control was managed with 0.05 mg/kg buprenorphine every 12 h. While, to examine the therapeutic effects of rGDF3 in WT mice upon CLP surgery, rGDF3 (100 μg/kg body weight), or BSA vesicle was injected into the tail vein 1 h after CLP. In order to assess the actual effect of rGDF3 on the survival of septic mice, antibiotics was not used in the CLP model. The animal survival rate was monitored every 6 h for 7 days after CLP operation. Whole blood and peritoneal lavage fluid (PLF) were harvested from these mice to measure bacterial burden, cytokines, and markers of organ injury at 20–24 h post-CLP surgery. Bacterial burden assay was performed as described previously (28).

### Lung Histology and Lung Injury Score

Lung tissues were collected from mice at 24 h post-CLP. All lungs were perfused *via* the heart, inflated and fixed with 10% buffered formalin for more than 2 days, then embedded in paraffin, and cut into 5-μm sections. Tissue sections were stained with hematoxylin and eosin (H&E). To evaluate the lung injury, 6–8 independent random lung fields were evaluated per mouse for neutrophils in alveolar spaces, neutrophils in the interstitial spaces, hyaline membranes, proteinaceous debris filling the airspaces, and alveolar septal thickening and weighted according to the relevance ascribed by the official American Thoracic Society workshop report on features and measurements of experimental acute lung injury in animals (29).

### Tissue Wet-to-Dry Weight Ratio

To quantify the spleen and lung edema, whole spleen and lung tissues were collected at 24 h post-CLP, rinsed to remove surface blood and patted dry, and the immediate weights of the samples were recorded as the wet weight. The tissues were air dried for 48 h at 60°C, and the weights were recorded as the dry weight. A wet/dry weight ratio for each individual mouse was calculated.

### Assessment of Liver and Kidney Injury

Serum levels of alanine aminotransferase (ALT), a biochemical marker of liver injury, were measured using an ALT assay kit (MyBioSource, Cat. # MBS264717). Serum levels of creatinine (Cr), a biochemical marker of kidney injury, were determined using a creatinine assay kit (MyBioSource, Cat. # MBS2504918), according to manufacturers' protocols.

### Measurement of Cytokines

Blood and PLF were collected at 20 h after CLP surgery and serum samples were prepared by centrifuged at 3,000 g for 15 min at 4°C. All serum was stored in −80°C until use. ELISAs were performed to measure the levels of TNF-α and IL-6 in sera and PLF using commercially available kits [Peprotech, Cat. # 900-K54 (TNF-α), Cat. # 900-K50 (IL-6)], according to the manufacturer's protocols.

### Cell Preparation

For the isolation of peritoneal macrophages (PMs), 5 mL PBS was used to lavage the abdominal cavity of euthanized mice for three times. The PLF was collected and centrifuged (2,000 rpm, 5 min). The cell pellet was then resuspended in 10 mL fresh prepared Dulbecco's modified Eagle medium (DMEM) media with 10% fetal bovine serum (FBS) and 1% penicillin/streptomycin. The cells were allowed to adhere to the substrate by culturing them for 4 h at 37°C. Non-adherent cells were removed by gently washing three times with warm PBS. Adherent cells were cultured for 18 h, and then used for experiments.

Bone marrow-derived macrophages (BMDMs) were isolated cultured/differentiated into macrophages as described previously (30). In brief, mice were terminally anesthetized, bone marrow from the tibias and femurs of mice was flushed out using cold sterile wash medium (DMEM), and filtered through 70 μm Nylon cell strainer. Subsequently, these mononuclear cells were centrifuged at 500 g for 5 min at room temperature (RT). The resulting cell pellet was re-suspended in complete culture medium (DMEM supplemented with 10% of L929 cell culture supernatant, 10% FBS, 1% penicillin/streptomycin solution, and 1% HEPES), and cultured in a humidified atmosphere of 5% CO_2_ at 37°C for 7 days, culture medium was changed on Day 4 of culture.

RAW264.7 macrophages (mouse macrophage cell line) were cultured in incubator at 37°C with 5% CO_2_. The cells grew in DMEM, containing 10% FBS and 1% penicillin/streptomycin.

### MTS Cell Proliferation Assay

BMDMs and RAW264.7 macrophages seeded into 96-well plates were treated with different rGDF3 doses for 18 h. Cell viability was measured by addition of 3-(4,5-dimethylthiazol-2-yl)-5-(3-carboxymethoxyphenol)-2-(4-sulfophenyl)-2H-tetrazolium (MTS, Promega, USA) for 1.5 h at 37°C. The absorbance was measured spectrophotometrically at 490 nm with a microplate reader (ELx800, Bio-Tek, USA) according to the manufacturer's protocols.

### Phagocytosis Assay With *Escherichia coli* BioParticles

To analyze the phagocytic capacity of RAW264.7 macrophages and BMDMs isolated from WT mice, cells were seeded into 96-well plates (4 × 10^4^ cells/well; or 1 × 10^5^ cells/well) or on coverslips in 24-well plates (5 × 10^4^ cells/well; or 2 × 10^5^ cells/well) in DMEM medium and allowed to adhere for 24 h. Cells were treated with BSA, rGDF3 (20 ng/mL), GW3965 (1 μmol/L) or GSK2033 (2 μmol/L) + rGDF3 (20 ng/mL) for 18 h, followed by the addition of pHrodo red *E. coli* BioParticles (Thermal Fisher Scientific, Cat. # P35361) diluted in medium according to the manual. Subsequently, cells were incubated with BioParticles for 1.5 h at 37°C. The fluorescence intensity was measured using a GloMax®-Multi Detection System (Promega). While cells cultured on coverslips were stained with CellMask™ Green Plasma Membrane Stain (Invitrogen, Cat. # C37608) as described in the manual. Then, cells were fixed with 4% paraformaldehyde (15 min RT). After washing three times with PBS, coverslips were mounted onto slides using a ProLong™ diamond antifade mountant reagent with DAPI (Invitrogen, Cat. # P36962). Subsequently, slides were imaged with a confocal LSM 710 (Carl Zeiss Microimaging, Jena, Germany). Images were recorded with ZEN (Black) and analyzed with ImageJ software (Wayne Rasband, National Institutes of Health, Bethesda, MD).

### Flow Cytometry

PMs isolated from WT mice were seeded into 6-well plates (8 × 10^5^ cells/well) in DMEM medium and allowed to adhere for 18 h. Cells were treated with BSA or rGDF3 (20 ng/mL) for 18 h, followed by the pHrodo red *E. coli* BioParticles according to the manual. Subsequently, cells were incubated with BioParticles for 1.5 h at 37°C. Then, cells were collected and fixed with 2% paraformaldehyde (15 min on ice). After washing with FACS buffer, Flow cytometry analysis was performed.

### Bacterial Phagocytosis and Killing Assay

To determine bactericidal activity, a classical CFU assay was conducted with minor modifications as described previously (31). Briefly, *E. coli* DH5 alpha were grown overnight in LB at 37°C. After that, bacteria were quantified, pelleted, and washed with PBS. BMDMs or RAW264.7 macrophages were placed in 12-well-culture plates and pre-incubated for 6 h to allow to adhere. Subsequently, cells were treated with BSA or rGDF3 (20 ng/mL) in antibiotic-free fresh medium for 18 h. Cells were infected with live *E. coli* at a multiplicity of infection (MOI) of 20 bacteria/cell ratio for 1 h at 37°C with 5% CO_2_. Fresh DMEM containing 100 μg/mL gentamicin was added and incubated for 30 min to kill extracellular bacteria. To determine internal bacteria, macrophages were washed two times with cold PBS and lysed. The cell lysate was diluted serially and plated on the LB plate to determine the colony-forming unit (CFU) count. To assess the bactericidal activity, cells were incubated for another 6 h. The number of bacteria remained after intracellular killing was determined by lysing cells and plating the cell lysate as described in above. The killing percentage was calculated by using the formula [(CFU count at 1 h) – (CFU count at 6 h)/(CFU count at 1 h)] × 100%.

### Isolation of RNA and Quantitative Real-Time PCR (qRT-PCR)

Total RNA was extracted from cultured cells using the RNeasy kit (Qiagen, Cat. # 217004) in accordance with the manufacturer's instructions. cDNA was synthesized from 0.5–1.0 μg total RNA using Superscript II Reverse Transcriptase (Invitrogen, Cat. # 18080044). Then, qRT-PCR was performed in triplicate with the ABI PRISM 7900HT sequence detection system (ABI) using SYBR green (Genecopoeia, Cat. # QP005). Relative mRNA levels were calculated and normalized to 18S rRNA. The primers were used as follows: CD5L forward 5'-TTGGAGAACAACTGTACCCATGGC-3', reverse 5'-AGGCTGAGGGAAAGGTGTCTAAAG-3'; Bcl2a1b forward 5'-GTTTCCAGTTTTGTGGCAGA-3', reverse 5'-CCCAGAACTGTCCTGTCATC-3'; MRPS18C forward 5'-GCATTTATGGAAGGCACATAAC-3', reverse 5'-TGGCAGGAACTTCACAAATAC-3'; 18S rRNA forward 5'-GCAATTATTCCCCATGAACG-3', reverse 5'-GGCCTCACTAAACCATCCAA-3'. Directional polyA RNA-sequencing was performed by the Genomics, Epigenomics, and Sequencing Core (GESC) at the University of Cincinnati.

### Immunofluorescence Staining

BMDMs treated with rGDF3 (20 ng/mL) and GW3965 (1 μmol/L) (DMSO used as control) were fixed on the coverslip, blocked with 1% BSA, 0.3% Triton X-100, 0.1% Tween 20 and incubated with primary antibody (LXRα, Invitrogen, Cat. # PA1-330) diluted in 1% BSA, 0.1% Tween 20 overnight at 4°C. After washing, BMDMs were incubated with secondary antibody at RT for 1 h. Thereafter, cells were washed three times with PBS and mounted with ProLong diamond Antifade Mounting medium with DAPI (Invitrogen, Cat. # P36962). Images were captured with Zeiss LSM710 LIVE Duo Confocal Microscope (Live Microscopy Core, University of Cincinnati).

### Statistical Analysis

Statistical calculations were performed with GraphPad Prism 7.0 software (GraphPad Software, Inc., USA). The experimental data were expressed as mean ± SEM. Differences between two groups were analyzed using unpaired two-tailed t-test and survival rates were analyzed using log rank test. *p* < 0.05 was considered to be statistically significant.

## Results

### Administration of rGDF3 Attenuates Polymicrobial Sepsis-Induced Mortality and Organ Injury in Mice

**Figure 1:**
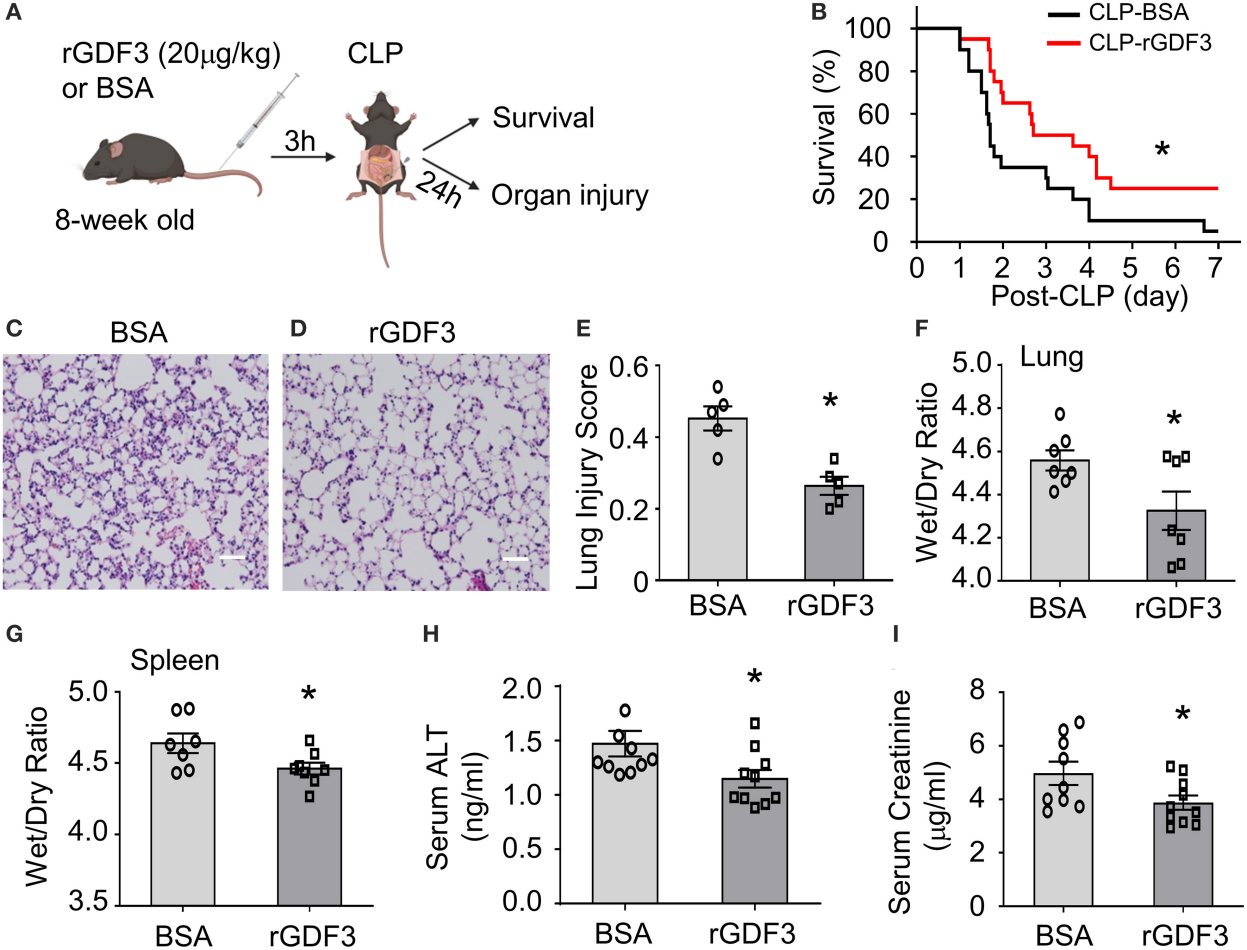
Administration of rGDF3 attenuates polymicrobial sepsis-induced mortality and organ injury in mice. **(A)** Graphic illustration of experimental design: WT mice were received rGDF3 (20 μg/kg, or BSA as control) through the tail-vein injection, followed by CLP surgery. **(B)** Kaplan–Meier survival curves were generated to compare mortality between 2 groups, significance was determined by log-rank (Mantel-Cox) test (*p < 0.05; *n* = 20 per group). **(C,D)** Representative images of lung sections with hematoxylin and eosin (H&E) staining from both **(C)** BSA- and **(D)** rGDF3-treated mice at 24 h after CLP surgery (Scale bar, 50 μm). **(E)** The lung injury scores were assessed as described in section Materials and Methods (*n* = 5). **(F,G)** The wet weight to dry weight ratios of **(F)** lung and **(G)** spleen in mice treated with BSA vesicle or rGDF3 at 24 h post-CLP were quantified (*n* = 7–8). **(H,I)** Serum levels of **(H)** alanine aminotransferase (ALT), a biochemical marker of liver injury, and **(I)** creatinine (Cr), a biochemical marker of kidney injury, in both groups were measured using ELISA. Data are representative of two independent experiments. Results are presented as mean ± SEM and analyzed by Student's *t-*test (*p < 0.05).

Our recent work showed that rGDF3 suppressed inflammatory response and improved cardiac function and survival in endotoxin-induced sterile inflammation mouse model. To explore whether rGDF3 could provide a preventive effect in response to polymicrobial sepsis, WT mice were received rGDF3 (20 μg/kg body weight) or BSA vesicle *via* the tail vein injection 3 h prior to CLP surgery (Figure 1A), and then mortality was monitored over 7 days. We observed that rGDF3-treated mice exhibited significantly higher survival rate than BSA-treated mice over a period of 7 days post-CLP (Figure 1B). Median survival was 76 h in rGDF3-treated group and 41 h in BSA-treated group (Figure 1B, *p* < 0.05). Next, we assessed organ injury in BSA- and rGDF3-treated mice at 24 h post-CLP by: (1) conducting histopathological analysis, (2) determining tissue edema, and (3) measuring biomarkers of organ damages in the serum. Firstly, we found that the administration of rGDF3 significantly rescued CLP-induced lung injury, as evidenced by the decrease in neutrophil infiltration, formation of hyaline membranes, thickness of alveolar wall, and the alveolar collapse in lung tissues collected from rGDF3-treated mice (injury score 0.264 ± 0.025), compared to those from BSA-treated mice (injury score 0.452 ± 0.034) (Figures 1C–E). In addition, rGDF3-treated mice exhibited lower ratio of wet weight/dry weight than that from BSA-treated mice (4.325 ± 0.089 vs. 4.558 ± 0.047) (Figure 1F). Similar results were observed for the wet weight/dry weight ratio of the spleen (4.462 ± 0.041 vs. 4.639 ± 0.069) (Figure 1G). Furthermore, we tested serum levels of alanine transaminase (ALT) and creatinine (Cr), biomarkers of liver injury and kidney damage, respectively. As shown in Figure 1H, I, the concentrations of both biomarkers were significantly lower in rGDF3-treated mice, compared to BSA-treated mice at 24 h post-CLP. Collectively, these data indicate that pre-administration of rGDF3 significantly decreases CLP-induced multi-organ injury in mice.

### Administration of rGDF3 Decreases Bacterial Burden and Cytokine Levels in Response to CLP

**Figure 2:**
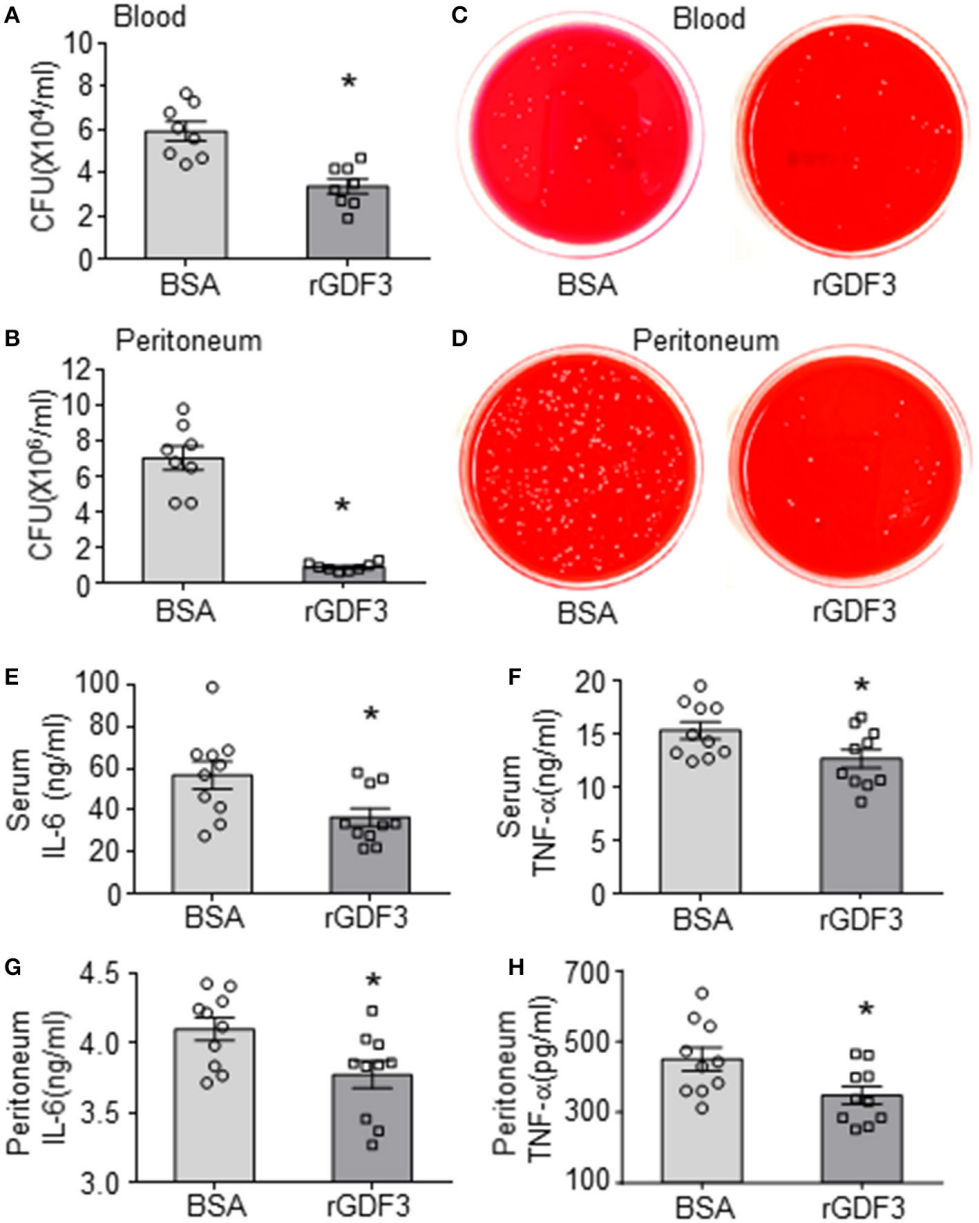
Administration of rGDF3 decreases bacterial burden and cytokine levels in response to CLP. **(A–D)** The bacterial burden in both blood **(A, C)** and PLF **(B, D)** were compared between BSA- and rGDF3-treated mice at 20 h after CLP surgery. **(E–H)** Cytokine levels in serum [**(E)**: IL-6, **(F)**: TNF-α] and PLF [**(G)**: IL-6, **(H)**: TNF-α] in mice were measured at 20 h after CLP surgery using ELISA assays (*n* = 9–10 per group). CFU, colony-forming unit. Data are representative of two independent experiments. All results are presented as mean ± SEM and analyzed by Student's *t-*test (*p < 0.05).

Since bacterial burden and inflammatory responses are important driving factors for the pathogenesis and progression of sepsis (1, 6, 7), we next sought to determine the effects of rGDF3 on these aspects. The bacterial burdens in the blood and PLF were examined at 20 h post-CLP surgery. As shown in Figures 2A,B, the rGDF3-treated group presented significantly lower bacterial load in blood and PLF compared to control group (BSA-treated). Representative images for CFUs in both blood and PLF were shown in Figures 2C,D. In parallel, results from ELISA analysis showed that the levels of inflammatory cytokines IL-6 and TNF-α were significantly suppressed by rGDF3 administration in both sera (Figures 2E,F) and PLFs (Figures 2G,H). Together, these results suggest that the beneficial effects of rGDF3 on survival and organ damage may be associated with its ability to control bacterial burden and inflammatory responses.

### rGDF3 Treatment Promotes Phagocytic and Bactericidal Activities of Macrophages

**Figure 3:**
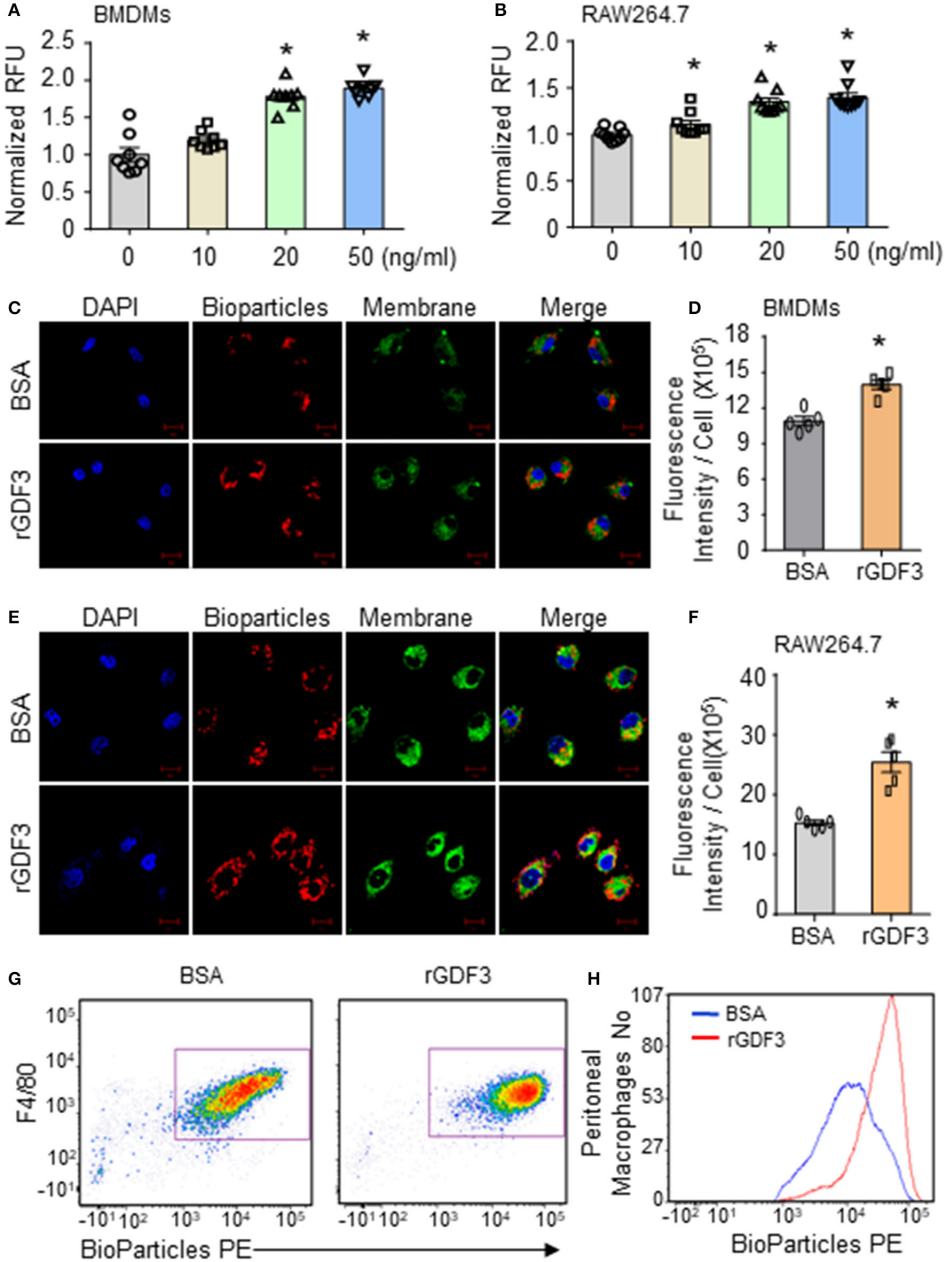
rGDF3 treatment promotes phagocytic activity of macrophages. **(A,B)** After stimulating BMDMs **(A)** and RAW264.7 macrophages **(B)** with different doses of rGDF3, the phagocytic capacity was assessed by adding Red fluorescence–conjugated pHrodo *E. coli* BioParticles. **(C–F)** Representative confocal images of phagocytosis assay in BMDMs **(C)** and RAW 264.7 macrophages **(E)** with *E. coli* BioParticles after rGDF3 treatment (scale bar, 10 μm). The mean fluorescence intensity in BMDMs **(D)** and RAW 264.7 macrophages **(F)** was quantified. **(G,H)** Representative flow cytometry plot **(G)** and histogram **(H)** showing PM (F4/80+) phagocytosis of *E. coli* BioParticles after rGDF3 treatment. Similar results were obtained in another separated experiment. All results are shown as mean ± SEM and analyzed by Student's *t-*test (*p < 0.05).

**Figure 4:**
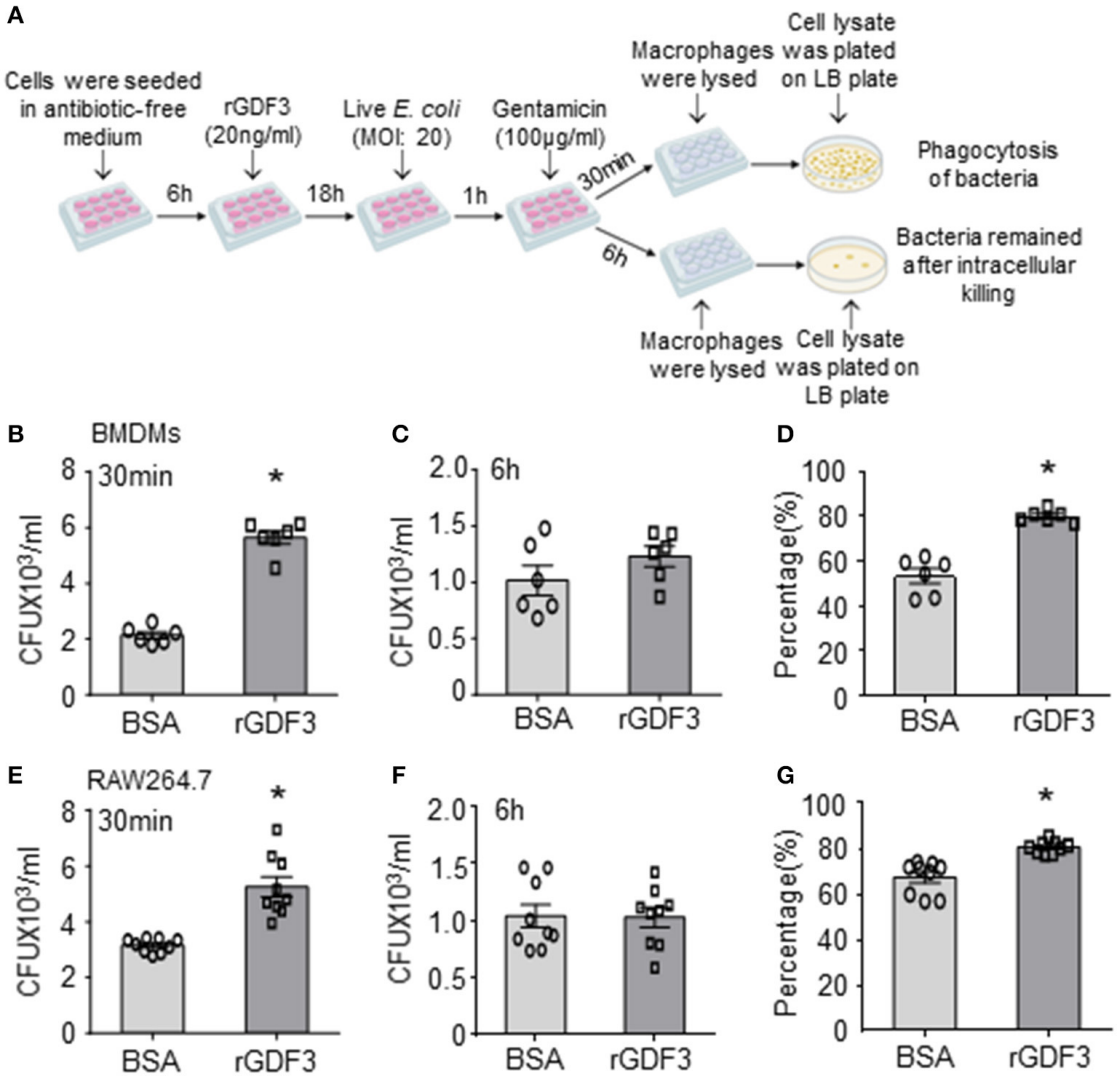
rGDF3 treatment promotes macrophage phagocytosis and intracellular killing of live bacteria. **(A)** Graphic illustration of experimental design: Gentamicin protection assay was used to test the phagocytic and bactericidal activities of BMDMs and RAW 264.7 macrophages using live *E. coli*. After 1 h of infection, Gentamicin was added to the cell culture medium. After 30 min, cell lysate was extracted with serial dilution, then plated on LB agar plates. The CFUs were measured as an indicator for phagocytosis capacity of BMDMs **(B)** and RAW 264.7 cells **(E)**. In addition, 6 h after the gentamicin was added, the CFUs isolated from BMDMs **(C)** and RAW 264.7 cells **(F)** were determined to assess the number of bacteria that remained inside macrophages. The killing percentages of BMDMs **(D)** and RAW264.7 macrophages **(G)** were calculated as described in section Materials and Methods (*n* = 6–9). Data are representative of three independent experiments. All results are shown as mean ± SEM and analyzed by Student's *t-*test (*p < 0.05).

Considering that macrophages play an essential role in bacterial clearance during polymicrobial sepsis (12), we next conducted bacterial phagocytosis and intracellular killing assays in three different sources of macrophages: (1) RAW264.7 cell line, (2) BMDMs, and (3) PMs isolated from WT mice. Firstly, we investigated the effect of rGDF3 on the cell viability with MTS assay. As shown in Supplementary Figures 1A,B, stimulation with different rGDF3 doses (10, 20, 50 ng/mL) did not affect the viability of BMDMs and RAW264.7 cells. Next, using the fluorescence-conjugated *E. coli* BioParticles, we found that treatment of macrophages with rGDF3 at doses of 20 and 50 ng/mL greatly increased the uptake of *E. coli* BioParticles in both BMDMs (Figure 3A) and RAW264.7 cells (Figure 3B). Of interest, rGDF3 at a lower dose (10 ng/mL) significantly enhanced phagocytosis of *E. coli* BioParticles in RAW264.7 macrophages but not BMDMs (Figure 3B). To further validate these results, the uptake of fluorescence-conjugated *E. coli* BioParticles was examined under confocal microscopy. We found that the intensity of red particles in rGDF3-treated BMDMs was significantly higher than that in control BSA-treated cells (Figures 3C,D). Similar findings were also observed in rGDF3-treated RAW264.7 cells (Figures 3E,F). In addition, flow cytometry analysis results further showed that rGDF3-treated PMs displayed higher content of red fluorescence (Figures 3G,H). Thus, these data generated from all three different analysis methods consistently indicate that rGDF3 is able to augment macrophage phagocytosis. Lastly, we assessed the phagocytic and bactericidal activities of BMDMs and RAW264.7 cells using live *E. coli*, as described in Figure 4A. We found that rGDF3 treatment significantly increased the bacterial uptake by 2.64-fold in BMDMs (Figure 4B) when compared to BSA-treated cells. The numbers of bacteria remained in BMDMs at 6 h after intracellular killing were shown in Figure 4C. Remarkably, rGDF3 stimulation could boost intracellular killing activity by 26.59% in BMDMs, compared to BSA-treated controls (Figure 4D). Similar findings were also observed in rGDF3-treated RAW264.7 cells, in which the uptake of bacteria and intracellular killing activity were significantly increased, compared to BSA-treated cells (Figures 4E–G). Taken together, these data indicate that treatment of macrophages with rGDF3 could improve phagocytosis and bactericidal activities.

### RNA Sequencing Analysis of Gene Expression Profile in BMDMs Treated With rGDF3

**Figure 5:**
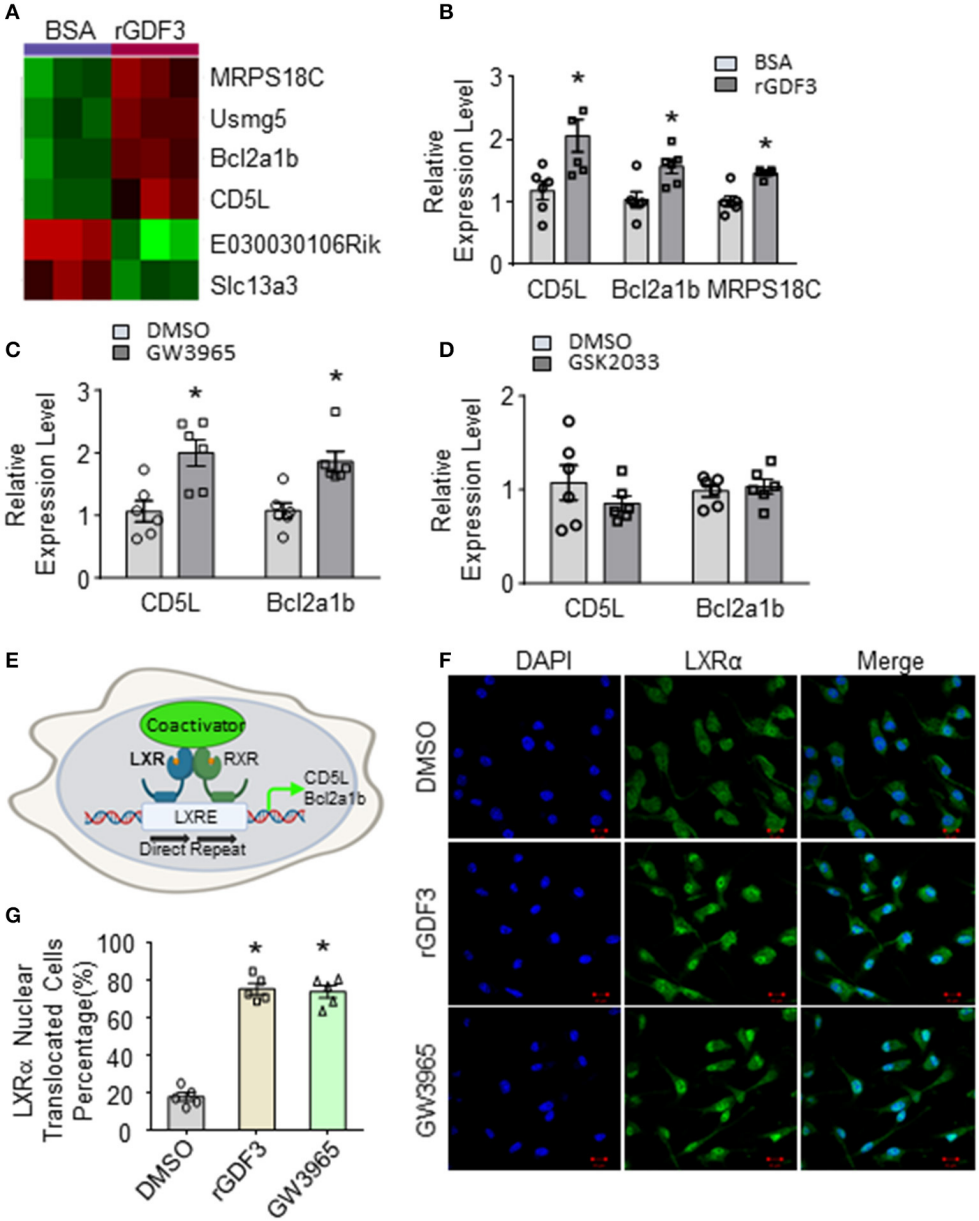
Gene expression profile in BMDMs treated with rGDF3 by high-throughput RNA sequencing. **(A)** Heatmap of the differentially expressed genes from BMDMs treated with BSA or rGDF3 (20 ng/mL) (*n* = 3). **(B)** The altered expression of CD5L, Bcl2a1b, and MRPS18C gene in rGDF3-treated BMDMs was validated by qRT-PCR (*n* = 6). **(C)** Expression of CD5L and Bcl2a1b in GW3965 (1 μmol/L)-treated BMDMs was determined by qRT-PCR (*n* = 6) at 18 h post-treatment. **(D)** Expression of CD5L and Bcl2a1b in BMDMs were measured at 18 h post-GSK2033 (2 μmol/L) treatment (*n* = 6). **(E)** Graphic scheme of the LXRα-CD5L signal cascade in macrophages. Representative images **(F)** and quantification **(G)** of immunofluorescence staining for LXRα (green) in BMDMs at 18 h after rGDF3 (20 ng/mL) or GW3965 (1 μmol/L) treatment (Scale bar, 10 μm; *n* = 5). Data are representative of two **(B–D,F,G)** independent experiments. All results are shown as mean ± SEM and analyzed by Student's *t*-test (*p < 0.05).

To identify potential mechanisms underlying the effect of rGDF3 in promoting macrophage phagocytosis of bacteria, we treated BMDMs with rGDF3 (20 ng/mL) or control BSA for 18 h, followed by isolation of total RNAs for RNA sequencing (RNA-seq). Unexpectedly, we identified only 6 differentially expressed genes, of which 4 were significantly upregulated and 2 were down-regulated (*p* < 0.05), as listed in Supplementary Table 1 and illustrated in Figure 5A. Of interest, among these 4 up-regulated genes, CD5L has been recently reported to stimulate the phagocytosis of bacteria in macrophages (32). CD5L, also referred to as apoptosis inhibitor 6 (Api6) or apoptosis inhibitor of macrophages (AIMs), is a soluble protein mainly produced by macrophages (32, 33). Importantly, the expression of CD5L is controlled by the activation of LXRα in macrophages (33). Indeed, another LXRα-controlled gene, Bcl2a1b (34), was also significantly upregulated in rGDF3-treated macrophages (Figure 5B). Therefore, we speculated that rGDF3 might enhance macrophage phagocytosis through activating LXRα pathway. To test this hypothesis, we treated BMDMs with GW3965, a potent LXRα agonist, followed by measuring the expression levels of CD5L and Bcl2a1b. qRT-PCR results showed that activation of LXRα by its agonist significantly increased mRNA levels of CD5L and Bcl2a1b, compared with control-treated macrophages (Figure 5C). In contrast, blockade of LXRα in BMDMs by its antagonist, GSK2033, offset rGDF3-mediated upregulation of CD5L and Bcl2a1b (Figure 5D). Given that the activation of LXRα is associated with its translocation to the nucleus (35, 36), as indicated in Figure 5E, we next determined whether rGDF3 could mimic the effect of LXRα agonist GW3965 on LXRα translocation, using immunofluorescence staining. We observed that the number of macrophages with nuclear translocation of LXRα was similar between rGDF3- and GW3965-treated groups (Figures 5F,G). Put together, these results demonstrate that rGDF3 could activate LXRα pathway in macrophages.

### Inhibition or Deficiency of LXRα Abolishes rGDF3-Mediated Effects on Macrophage Phagocytosis and Bacterial Killing Activities

**Figure 6:**
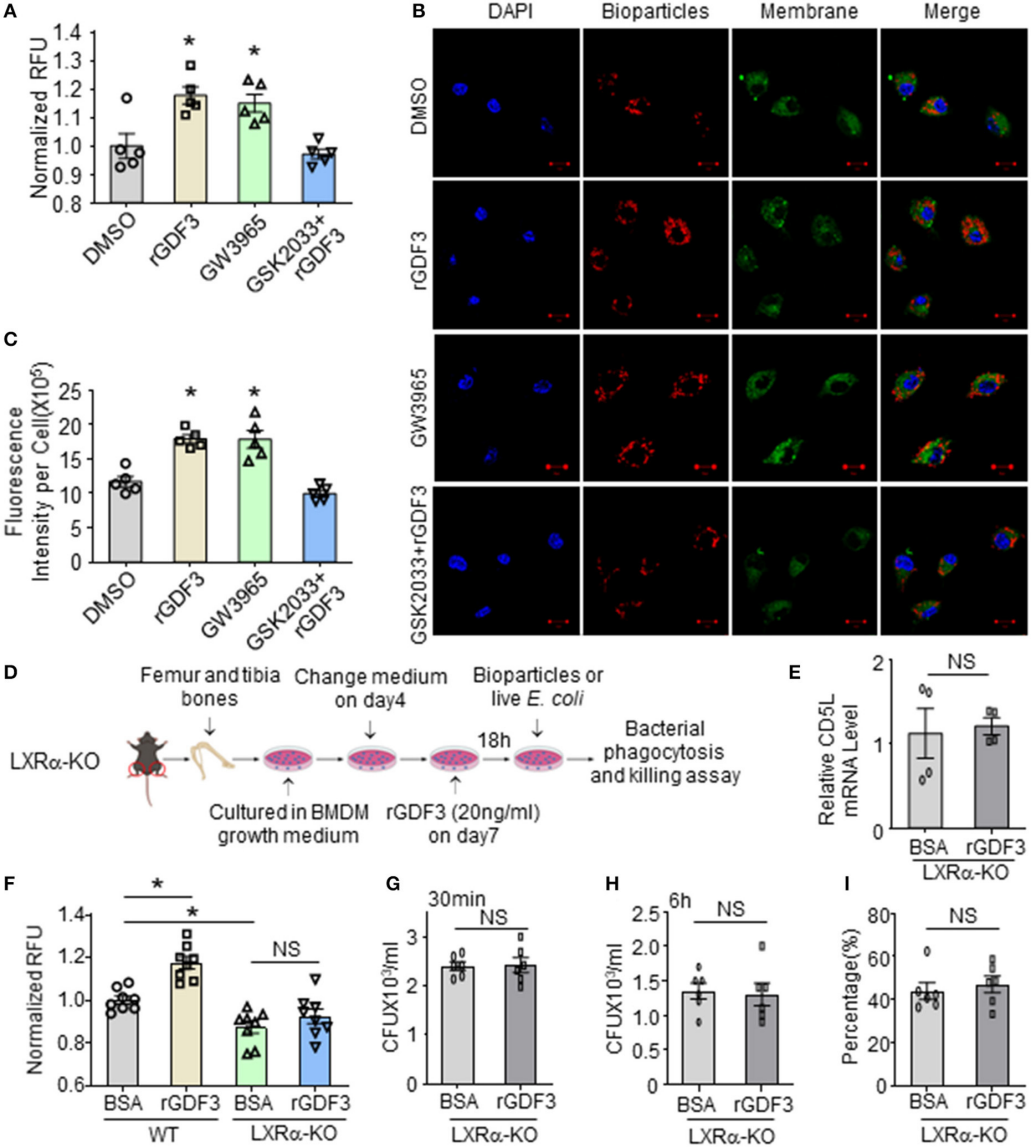
Inhibition or deficiency of LXRα abolishes rGDF3-mediated effects on macrophage phagocytosis and bacterial killing activities. **(A)** BMDMs were treated with DMSO (0.005%, vesicle control), rGDF3, GW3965, and GSK2033+rGDF3 for 18 h, then phagocytic capacity was assessed using red *E. coli* BioParticles. **(B)** Representative confocal images of phagocytosis assay in BMDMs treated with DMSO, rGDF3, GW3965, and GSK2033 + rGDF3 with red *E. coli* BioParticles (Scale bar, 10 μm), and **(C)** their quantifications of the mean fluorescence intensity in each group. **(D)** Graphic scheme of experimental design for BMDMs isolated from LXRα-KO mice and following treatment. **(E)** The altered expression of CD5L gene in rGDF3-treated BMDMs isolated from LXRα-KO mice was validated by qRT-PCR (*n* = 4). **(F)** Phagocytic capacity of WT- and LXRα-KO-BMDMs after rGDF3 treatment was assessed by adding *E. coli* BioParticles. **(G–I)** Gentamicin protection assay was used to test the phagocytic and bactericidal activities of WT- and LXRα-KO-BMDMs. **(G)** The CFUs were measured as an indicator for phagocytosis capacity of BMDMs. **(H)** 6 h after the gentamicin was added, the bacterial residue remained inside macrophages were assessed. **(I)** The killing percentages of LXRα-KO BMDMs were calculated (*n* = 6). Similar results were obtained in other two independent experiments. All results are shown as mean ± SEM and analyzed by Student's *t-*test (*p < 0.05).

Next, we determined whether the activation of LXRα is responsible for rGDF3-mediated increase of bacterial clearance by macrophages. To this end, we pre-treated BMDMs with LXRα antagonist, GSK2033, followed by addition of rGDF3 (20 ng/mL). LXRα agonist (GW3965, 1 μmol/L) and rGDF3 alone were used as controls. Subsequently, phagocytosis of *E. coli* BioParticles was analyzed. We observed that treatment with both LXRα agonist and rGDF3 alone could significantly enhance BMDMs phagocytosis of *E. coli* BioParticles (Figure 6A). However, rGDF3 failed to improve phagocytosis of *E. coli* BioParticles in BMDMs pretreated with GSK2033 (Figure 6A). These results were also validated with confocal microscopy, as shown in Figures 6B,C that the intensity of red fluorescence in GW3965- and rGDF3-treated macrophages was significantly higher than in DMSO-treated cells. Meanwhile, the effects of rGDF3 on phagocytosis of *E. coli* BioParticles were blunted when BMDMs were pre-treated with LXR antagonist GSK2033, showing similar fluorescence intensity to the DMSO group (Figures 6B,C). Lastly, we isolated BMDMs from LXRα-KO mice (Figure 6D) and assessed CD5L expression by qRT-PCR after the treatment of BSA or rGDF3. As expected, rGDF3-treatment failed to increase the expression levels of CD5L in LXRα-KO BMDMs (Figure 6E). Furthermore, while treatment of LXRα-KO BMDMs with rGDF3 did not improve phagocytosis, loss of LXRα in macrophages significantly reduced the uptake of *E. coli* BioParticles under basal conditions (Figure 6F). Using living bacteria, we also observed that rGDF3-mediated enhancement of bacterial entry and intracellular killing was offset by knockout of LXRα (Figures 6G–I). Please note here that the entry of living bacteria into BSA-treated LXRα-KO cells (Figure 6G) appeared similar as BSA-treated WT cells presented in Figure 4B. This discrepancy may be ascribed to different batch of living bacteria. Indeed, higher number of living bacteria remained in LXRα-KO cells (Figure 6H), compared to WT-cells (Figure 4C). Accordingly, the percentage of intracellular killing in LXRα-KO cells (43%, Figure 6I) was lower than WT-cells (54%, Figure 4D) under basal conditions. Collectively, these results indicate that the activation of LXRα pathway is essential for rGDF3 to stimulate macrophages phagocytosis and killing of bacteria.

### Therapeutic Effects of rGDF3 in WT Mice Upon CLP Surgery

**Figure 7:**
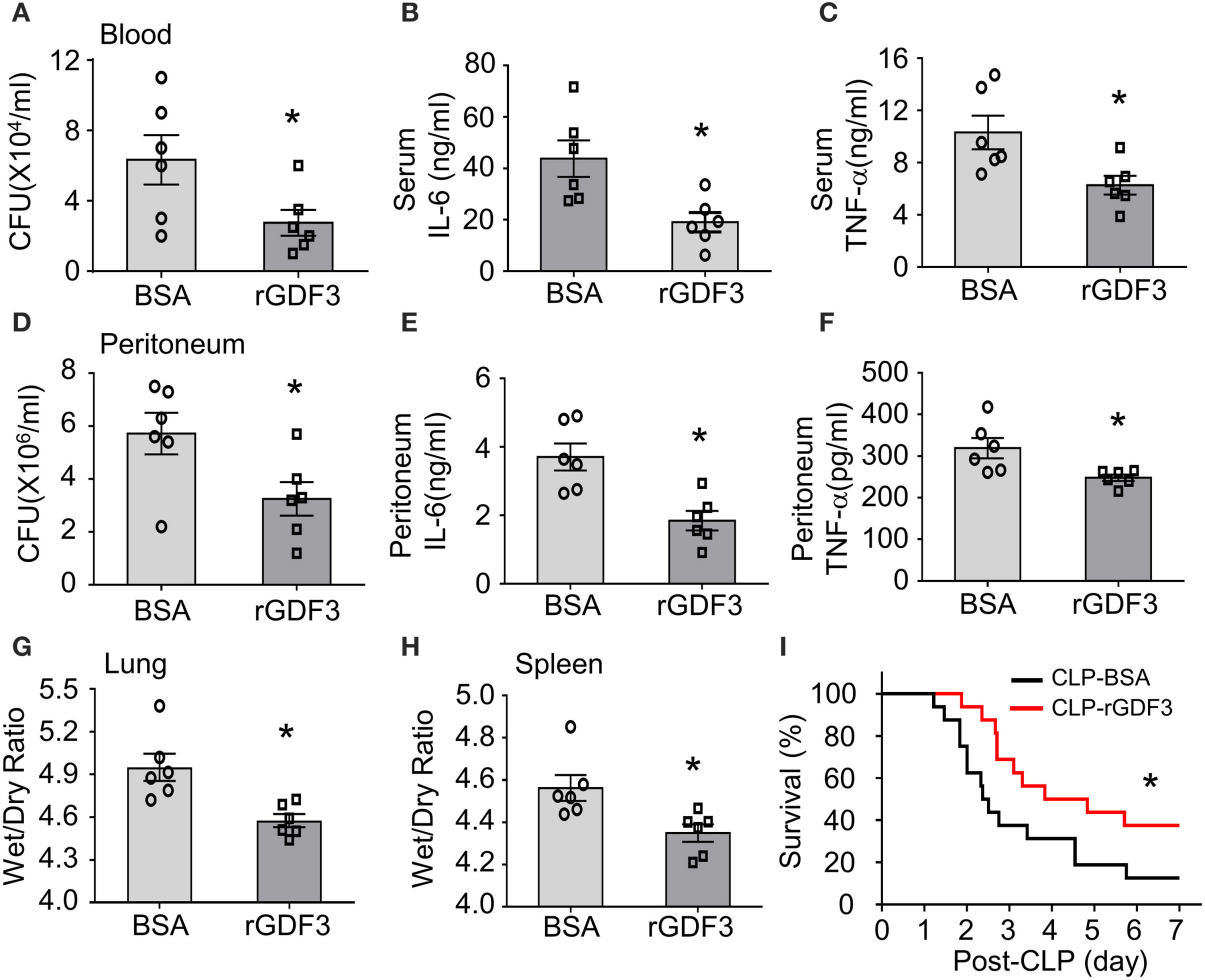
Therapeutic effects of rGDF3 in WT mice upon CLP surgery. A single dose of rGDF3 (100 μg/kg) or BSA vehicle was injected at 1 h post-CLP. **(A)** The bacterial burden in blood was compared between BSA- and rGDF3-treated mice at 24 h after CLP surgery. **(B,C)** Cytokine levels in sera [**(B)**: IL-6, **(C)**: TNF-α] of BSA- and rGDF3-treated mice were measured at 24 h post-CLP surgery using ELISA kits (*n* = 6). **(D)** The bacterial burden in PLF was compared between BSA- and rGDF3-treated mice at 24 h post-CLP surgery. **(E,F)** Cytokine levels in PLF [**(E)**: IL-6, **(F)**: TNF-α] of BSA- and rGDF3-treated mice were measured at 24 h post-CLP surgery (*n* = 6). **(G,H)** The wet weight to dry weight ratios of lung **(G)** and spleen **(H)** in BSA- and rGDF3-treated mice at 24 h post-CLP were quantified (*n* = 6). Similar results were obtained in another separated experiment. All results are presented as mean ± SEM and analyzed by Student's *t-*test (*p < 0.05). **(I)** Kaplan–Meier survival curves were generated to compare mortality between two groups, significance was determined by log-rank (Mantel-Cox) test (*p < 0.05; *n* = 16).

To further determine whether rGDF3 had any therapeutic effects *in vivo* against polymicrobial sepsis, we injected WT mice with a single dose of rGDF3 (100 μg/kg) or BSA vehicle at 1 h post-CLP surgery, followed by a series of experiments for the analysis of bacterial burden, cytokine levels, organ injury and mortality. We observed that post-administration of rGDF3 into CLP-mice significantly reduced bacterial burdens in the blood (Figure 7A) and PLF (Figure 7D) to a greater degree, compared to BSA-treated control mice. In addition, we found that the pro-inflammatory cytokines (i.e., IL-6 and TNF-α) levels in both serum (Figures 7B,C) and PLF (Figures 7E,F) were significantly lower in rGDF3-treated mice than controls. Furthermore, the ratios of wet/dry weight in lungs (Figure 7G) and spleens (Figure 7H) collected from rGDF3-treated CLP-mice were significantly reduced, compared to those samples from BSA-treated controls. Lastly, post-administration of rGDF3 into CLP-mice significantly improved survival outcome, as evidenced by 31.25% of rGDF3-treated mice (*n* = 16) survived at day 7 post-CLP, when only 6.25% of BSA-treated mice survived (*n* = 16). Meanwhile, the median survival was longer in rGDF3-treated mice than that in BSA-treated group (104 *vs*. 59 h) (Figure 7I, *p* < 0.05). Collectively, these data indicate that post-administration of rGDF3 could increase bacterial clearance, reduce inflammatory response, attenuate organ injury and improve animal survival in CLP-induced septic mice.

## Discussion

**Figure 8:**
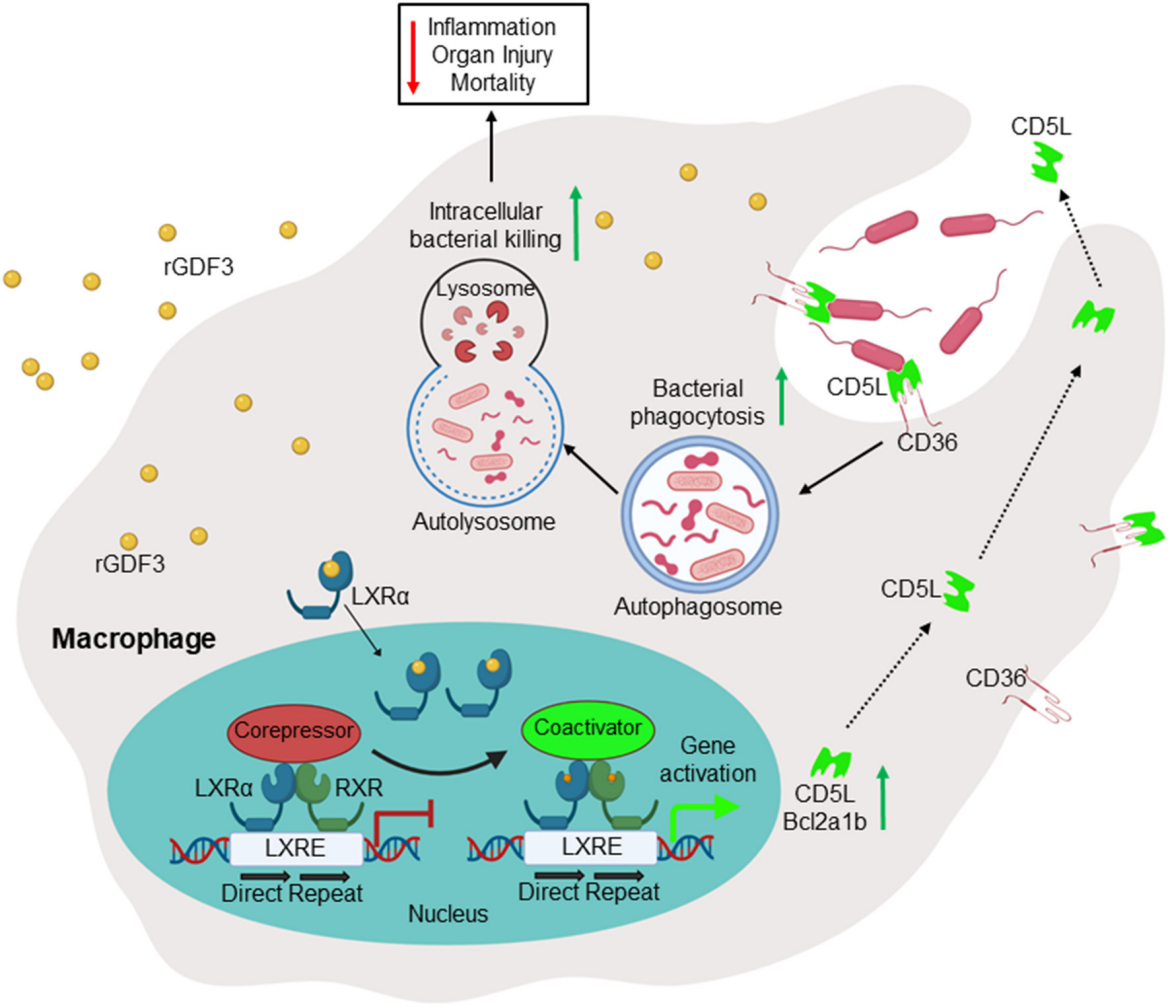
Scheme depicting that administration of GDF3 into septic mice improves survival *via* enhancing macrophage phagocytosis, which is mediated by activation of the LXRα-CD5L pathway.

It is well-recognized that impaired macrophage phagocytosis and intracellular killing of bacteria is the major culprit for insufficient antibacterial defense and hyper-inflammation in septic patients (37). In the present study, we showed that rGDF3 was able to increase bacteria uptake and killing by macrophages through activating LXRα pathway (Figure 8). Furthermore, either pre- or post-administration of rGDF3 significantly decreased polymicrobial sepsis-induced mortality in mice. This preferable outcome was associated with greatly reductions in systemic and local bacterial burden, inflammatory responses and multiple organ injury in CLP mice.

As one of the most important innate immune defenses, the phagocytosis and internal killing of pathogens by phagocytic cells is the first-line defense during infection due to its immediate response to pathogens. In patients, the reduced phagocytic activity during the first 24 h after admission has been identified as a positive predictor for mortality (38). Therefore, as “rapid response team,” macrophages are vital to host defense against microorganisms due to their ability in internalizing and digesting bacteria. Consistently, recent studies demonstrate that tissue-resident macrophages prevented neutrophil influx, neutrophil-mediated monocyte infiltration and inflammatory damage through prostrating their membranes to cloaking the minor tissue damage (39). Furthermore, we and others recently showed that enhancing the phagocytosis and bacterial killing activity of macrophage could improve animal survival in response to the polymicrobial sepsis (11, 40, 41). Along this line, our present study defined rGDF3 as a novel protector for enhanced clearance of bacteria by macrophages, leading to higher survival rate in response to the polymicrobial sepsis.

GDF3 was initially identified as a member of TGF-β superfamily to function in the early embryonic development (16–18). Prior work has shown that TGF-β signaling involved alternatively activation of macrophages (42). Our recent study demonstrated that patients with sepsis exhibited significantly higher levels of serum GDF3 as a compensatory mechanism in response to septic shock (26), suggesting the potential role of GDF3 in treating sepsis. In line with these findings, we observed that rGDF3 treatment could enhance phagocytosis and killing of bacteria in different kinds of macrophages, including BMDMs, RAW264.7 macrophages, and PMs. Furthermore, using a clinical-relevant polymicrobial sepsis model, we found that the administration of rGDF3 either before or after CLP surgery was able to improve survival and suppress inflammatory responses, which was associated with the reduced organ injuries.

The Liver X Receptors (LXRα and LXRβ) belong to nuclear receptors that regulate transcription of genes for cholesterol metabolism, cholesterol transport, and lipogenesis (43). Unlike LXRβ that is ubiquitously expressed, LXRα is dominantly expressed in liver and macrophages (44, 45). Interestingly, the present study identified that rGDF3 markedly stimulated the nuclear translocation of LXRα in macrophages. It is important to note here that previous studies implicated the essential role of LXR pathway for the expression of genes directly involved in antimicrobial responses, such as CD5L, which was reported to stimulate macrophage phagocytosis of bacteria (32, 33). In addition, LXR-deficient mouse models are more susceptible to bacterial-induced illness, and pharmacological treatment with LXR agonist improved clinical signs associated with bacterial infection (46). In line with these reports, the present study revealed that rGDF3 treatment significantly upregulated CD5L expression in macrophages and augmented macrophage phagocytosis. By contrast, the pretreatment of macrophages with LXRα antagonist (GSK2033) abolished the effects of rGDF3 on the expression of CD5L and macrophage phagocytosis. Consistently, our *ex vivo* results also showed that rGDF3 treatment failed to enhance phagocytosis and bacterial killing activities of BMDMs isolated from LXRα-KO mice. These results suggest that rGDF3-mediated macrophage phagocytosis is dependent on the activation of LXRα. Nonetheless, future studies should focus on the detailed mechanisms behind the interaction of rGDF3 and LXRα.

At present, CD5L has been well-studied in the regulation of antimicrobial response (32, 33, 47–52). CD5L is a macrophage-derived soluble protein belonging to the scavenger receptor cysteine-rich domain superfamily (SRCR-SF) (47). Like other SRCR-SF members (e.g., MARCO, SR-AI), CD5L exhibits pattern recognition and physically interacts with Gram-negative and -positive bacteria *via* directly binding to LPS, LTA, or peptidoglycan (48). More interestingly, recent studies further showed that CD5L could be transported into early endosomes and internalized by macrophages through CD36, a bona fide cell-surface receptor for CD5L (47, 48). Importantly, CD36 also recognizes bacterial cell-wall components and involves phagocytosis (49). In addition, the complex of CD5L/CD36 is a strong autophagy inducer in macrophages that may enhance intracellular killing of bacteria through autolysosomes (50–52). Therefore, the mechanism underlying rGDF3-induced phagocytosis could be through the activation of LXRα, leading to increased CD5L generation and consequently, CD5L works together with CD36 to recruit bacteria for phagocytosis (Figure 8). Given that a subset of small GTP-binding proteins (e.g., Rap1, Arf6, Rho) are well-known to control and coordinate the successive steps of the bacterial phagocytic process by macrophages (53–55), future investigations would be needed to address whether there is any crosstalk between the GDF3-LXRα-CD5L axis and the small GTP-binding proteins in the phagocytosis of bacteria by macrophages.

## Conclusions

Our data presented in this study for the first time demonstrate that, through activating the LXRα-CD5L pathway, GDF3 plays a critical role in enhancing phagocytosis and bactericidal function in macrophages. Pre- and post-administration of rGDF3 could attenuate polymicrobial sepsis-induced inflammation, organ damage, and mortality in mice. Therefore, GDF3 may be of great interest as a potential drug target or mediator to improve clinical outcomes for sepsis patients.

## Data Availability Statement

The raw data supporting the conclusions of this article will be made available by the authors, without undue reservation.

## Ethics Statement

The animal study was reviewed and approved by University of Cincinnati Animal Care and Use Committee.

## Author Contributions

PW designed and performed experiments, analyzed data and wrote the manuscript. XM designed experiments, helped to conduct flow cytometry experiments, and critically reviewed the manuscript. HZ, YL, LW, S-NC, and XW assisted with various experiments and critically reviewed the manuscript. VW and BZ helped to sacrifice LXRα-KO mice and collect samples. TP and CW helped with experimental design, data analysis, and critically reviewed the manuscript. G-CF analyzed results, reviewed/edited manuscript, provided financial and administrative support, and gave final approval of the manuscript. All authors contributed to the article and approved the submitted version.

## Conflict of Interest

The authors declare that the research was conducted in the absence of any commercial or financial relationships that could be construed as a potential conflict of interest.
