# Supplementary material for: Administration of GDF3 Into Septic Mice Improves Survival via Enhancing LXRα-Mediated Macrophage Phagocytosis
Source: Front Immunol. 2021 Feb 17;12:647070. doi: 10.3389/fimmu.2021.647070 (PMC7925632; doi:10.3389/fimmu.2021.647070)
Supplement: Supplementary file 1 [file Data_Sheet_1.docx]

Supplementary Material

## Supplementary Figure S1


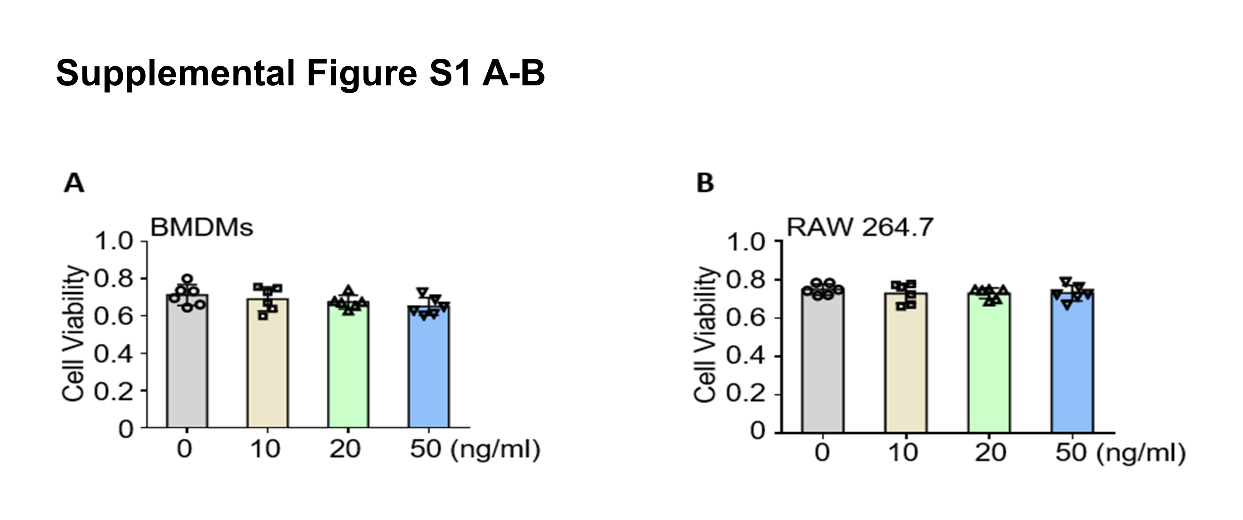


**Supplementary Figure S1.** **Cell viability analysis of macrophages upon the treatment of different doses of rGDF3 using MTS assay.**  (A and B) Cell viability was measured in both BMDMs (A) and RAW264.7 (B) treated with different doses of rGDF3.

## Supplementary Table S1

**Supplementary Table S1: RNA-seq analyses of the gene expression in BSA- or rGDF3-treated BMDMs**

| geneid | symbol | name | Base Mean | Base Mean Group BSA | Base Mean Group GDF3 | Fold-Change | log2 Fold Change | P val | P adj | GDF3-1 | GDF3-2 | GDF3-3 | BSA1 | BSA2 | BSA3 |
| --- | --- | --- | --- | --- | --- | --- | --- | --- | --- | --- | --- | --- | --- | --- | --- |
| 12045 | Bcl2a1b | B cell leukemia/lymphoma 2 related protein A1b | 2261.818 | 2099.885 | 2423.751 | 1.156371 | 0.209604 | 3.38E-06 | 0.034032 | 139.6668 | 141.2081 | 137.348 | 117.1713 | 122.9505 | 124.033 |
| 11801 | Cd5l | CD5 antigen-like | 10393.72 | 9639.819 | 11147.63 | 1.158655 | 0.212451 | 5.35E-06 | 0.034032 | 228.9331 | 251.8073 | 240.7188 | 205.6878 | 210.6201 | 210.7093 |
| 68735 | Mrps18c | mitochondrial ribosomal protein S18C | 657.315 | 602.962 | 711.6679 | 1.181957 | 0.241177 | 3.70E-05 | 0.082936 | 64.26935 | 62.41659 | 60.20109 | 50.77463 | 54.04491 | 54.31552 |
| 66477 | Usmg5 | upregulated during skeletal muscle growth 5 | 907.6504 | 843.0049 | 972.2958 | 1.154961 | 0.207844 | 3.91E-05 | 0.082936 | 127.7427 | 124.2344 | 123.9101 | 106.6444 | 112.0428 | 108.8687 |
| 319887 | E030030I06Rik | RIKEN cDNA E030030I06 gene | 55.87979 | 69.60818 | 42.1514 | 0.606031 | -0.72254 | 3.12E-05 | 0.082936 | 0.925516 | 0.705314 | 0.793566 | 1.360999 | 1.363872 | 1.299445 |
| 114644 | Slc13a3 | solute carrier family 13 (sodium-dependent dicarboxylate transporter), member 3 | 1941.783 | 2076.365 | 1807.201 | 0.87194 | -0.1977 | 2.59E-05 | 0.082936 | 23.94689 | 25.04154 | 24.90985 | 27.54997 | 29.61473 | 28.16937 |
